# Comparison of Phenolic Metabolites in Purified Extracts of Three Wild-Growing *Herniaria* L. Species and Their Antioxidant and Anti-Inflammatory Activities In Vitro

**DOI:** 10.3390/molecules27020530

**Published:** 2022-01-14

**Authors:** Solomiia Kozachok, Joanna Kolodziejczyk-Czepas, Svitlana Marchyshyn, Krzysztof Kamil Wojtanowski, Grażyna Zgórka, Wieslaw Oleszek

**Affiliations:** 1 Department of Biochemistry and Crop Quality, Institute of Soil Science and Plant Cultivation—State Research Institute, Czartoryskich 8, 24-100 Puławy, Poland; wieslaw.oleszek@iung.pulawy.pl; 2 Department of General Biochemistry, Faculty of Biology and Environmental Protection, University of Lodz, Pomorska 141/143, 90-236 Lodz, Poland; 3 Department of Pharmacognosy and Medical Botany, I Horbachevsky Ternopil National Medical University, Maidan Voli 1, 46001 Ternopil, Ukraine; marchyshyn@tdmu.edu.ua; 4 Department of Pharmacognosy with the Medicinal Plant Garden, Medical University of Lublin, 1 Chodzki St., 20-093 Lublin, Poland; krzyszofkamilw@gmail.com (K.K.W.); gzgorka@pharmacognosy.org (G.Z.)

**Keywords:** *Herniaria glabra* L., *H. polygama* J. Gay., *H. incana* Lam., Caryophyllaceae, LC-MS, phenolic metabolites, antioxidant, anti-inflammatory, cyclooxygenase, peroxynitrite

## Abstract

The work is aimed at phytochemical characterization and In Vitro evaluation of antioxidant actions, anti-inflammatory effects, and cytotoxicity of purified extracts from three rupturewort (*Herniaria* L.) species, i.e., *Herniaria glabra* (HG), *H. polygama* (HP), and *H. incana* herb (HIh). The total phenolic content established in the purified extracts (PEs) of HIh, HP, and HG was 29.6, 24.0, and 13.0%, respectively. Thirty-eight non-saponin metabolites were identified using LC-HR-QTOF-ESI-MS; however, only 9 were common for the studied *Herniaria* species. The most abundant phenolic compound in HG-PE was narcissin (7.4%), HP-PE shared 3 major constituents, namely *cis*-2-hydroxy-4-methoxycinnamic acid 2-*O*-β-glucoside (*cis*-GMCA, 5.8%), narcissin (5.4%), and rutin (5.3%). Almost half of HIh phenolic content (14.7%) belonged to oxytroflavoside A (7-*O*-methylkaempferol-3-*O*-[3-hydroxy-3-methylglutaryl-(1→6)]-[α-rhamnopyranosyl-(1→2)]-β-galactopyranoside). Antioxidant properties of the *Herniaria* PEs were evaluated employing an experimental model of human blood plasma, exposed to the peroxynitrite-induced oxidative stress. The assays demonstrated significant reduction of oxidative damage to protein and lipid plasma components (estimated by measurements of 3-nitrotyrosine, protein thiol groups, thiobarbituric acid-reactive substances), and moderate protection of its non-enzymatic antioxidant capacity. Anti-inflammatory properties of the *Herniaria* PEs were evaluated In Vitro as inhibitory effects against cyclooxygenases (COX-1 and -2) and concanavalin A-induced inflammatory response of the peripheral blood mononuclear cells (PBMCs). None of the studied plants showed inhibitory effects on COXs but all purified extracts partly reduced the release of interleukin 2 (IL-2) and tumor necrosis factor-alpha (TNF-α) from PBMCs, which suggested their prospective ability to up-regulate inflammatory response of the cells. The purified extract from *H. glabra* turned out to be the most efficient suppressor of PBMCs’ inflammatory response. Additionally, cytotoxicity of purified *Herniaria* extracts on PBMCs was ruled out based on In Vitro studies.

## 1. Introduction

Aerial parts of some *Herniaria* spp. from the family Caryophyllaceae are well documented herbal sources of medicinal substances used for the treatment of urolithiasis and prevention of recurrent kidney stones [1,2]. Their safety and therapeutic effects were confirmed in official monographs published by the European Medicines Agency (EMA) [3], and Austrian Pharmacopoeia [4].

The prevalence rate for urinary stones is continuously rising in countries with a high standard of life across the world from 1 to 20% (database from 2001 to 2007) [5,6]. The highest number of cases was reported in Saudi Arabia. It is well documented that the countries with hot, desert climates have the superior risk of urinary tract stone occurrence [7]. Climate changes (global warming), sedentary lifestyle, dietary habits (consumption of processed food, high content of animal proteins, sodium, sugars, and reduced amount of water) are believed to have a major contribution to an increasing stone prevalence [8]. Moreover, many sweetened drinks (Pepsi^®^, Coca-Cola^®^, noncola soda) contain phosphates, sugars, artificial sweeteners promoting the formation of calculi [9].

Pathophysiology of stone formation is characterized by insufficient urine volume and supersaturation of the urine with stone-forming constituents such as lithogenic ions (e.g., Ca^2+^, NH_4_^+^, C_2_O_4_^2−^, PO_4_^3−^, C_5_H_3_N_4_O_3_^−^ (urate), CO_3_^2−^), as well as a low concentration of stone inhibitor ions (Mg^2+^, C_6_H_5_O_7_^3−^ (citrate)).

A process of crystallization from solution has the following phases: nucleation, aggregation, and crystal growth [10]. In the 1st step, lithogenic crystals bind to the renal cells and stay there. Crystals tend to stick easily to the damaged side of the membrane [11]. Oxidative stress—the result of an imbalance between reactive oxygen species (ROS) and antioxidant capacity, plays a key role in the initiation and progression of damage to kidney cells. The renal deposits may disturb the electron transport chain in mitochondria, thereby triggering the release of mitochondrial ROS and the development of oxidative stress [8]. In addition, crystal formation, accompanied by free radical damage, stimulates the kidney cells to produce and release of inflammatory mediators. As a result, an acute inflammatory response develops that further exacerbates cell damage [7]. The next steps in stone formation are aggregation and growth, during which urine crystals adhere and bind to each other through an organic matrix. The latter contains proteins, lipids, polysaccharides, cellular debris, and whole cells, and makes up about 5% of the stone mass. It acts as a binding agent by increasing heterogeneous nucleation and aggregation of crystals [7,10,12]. Therefore, to effectively treat and prevent urolithiasis, it is important to use a combination of agents with antiurolithic, antioxidant, and anti-inflammatory properties.

In the previous study by Kozachok et al. [2], the possible antiurolithic mechanism of the main specific metabolites from *Herniaria glabra* L.—herniariasaponins, their role in stone recurrence, as well as detailed chemical characteristics of the mentioned metabolites were described. Herein, antioxidant and anti-inflammatory potential of purified *Herniaria* spp. extracts were characterized in the experimental system of human blood plasma exposed to the peroxynitrite-induced oxidative stress and inflammatory activities including cyclooxygenases (COX-1 and -2) inhibitor screening and the response of human peripheral blood mononuclear cells (PBMCs), respectively. Moreover, the cytotoxicity of the studied objects was examined towards PBMCs. The studied *Herniaria* spp. were *H. glabra* L. (HG) and *H. polygama* J. Gay (HP) whole plants, and *H. incana* Lam. herb (HIh, syn. *H. besseri* Fisch. ex Hornem). There is a large amount of evidence indicating that phenolic compounds are closely associated with, and responsible for antioxidant and anti-inflammatory activities [13]. Furthermore, in our previous studies, In Vitro single isolates from HG such as narcissin, licoagroside B, apiorutin as well as rutin, exhibited moderate antioxidant activity [14], and phenolic fractions from HIh and HP showed profound antioxidant and anti-inflammatory effects [15]. Thus, the detailed phytochemical characterization and quantification of phenolic metabolites in the purified extracts of HG, HP, and HIh were performed using UHPLC-DAD-QTOF-ESI-HRMS (ultra-high-performance liquid chromatography-diode array detector quadrupole time-of-flight electrospray ionisation high-resolution mass spectrometer) and UPLC-DAD-TQD-MS (TQD, tandem triple-quadrupole) respectively. This is the first study that compares non-triterpenoid profiles of *Herniaria* spp. and their antioxidant and anti-inflammatory effects In Vitro.

## 2. Results and Discussion

### 2.1. Phytochemical Profiles and the Content of Individual Phenolics in HG, HP, and HIh Purified Extracts

Water-methanolic *Herniaria* spp. extracts were defatted and purified from sugar and low molecular organic acids using RP-SPE (reversed-phase-solid phase extraction). The obtained purified extracts (PEs) containing *Herniaria* specific metabolites (HSM) were subjected to LC-MS analyses and bioassay tests. The major constituents of HSM comprised herniariasaponins [2] and non-saponin compounds. To estimate the content of the aforementioned compounds in the particular PEs, gel permeation chromatography (GPC) with evaporative light scattering detection (ELSD) was applied. GPC has the advantage of effectively separating triterpene glycosides (with MW > 1000 g/mol) from polyphenolic compounds, especially those with low MW [16]. Moreover, ELSD and charged-aerosol detector (CAD) are concentration sensitive and can be used to quantify analytes that lack UV chromophores or ionizable groups. They are suitable for saponin detection, unlike semi-volatile compounds [17]. As a final result, HG-PE, HP-PE, and HIh-PE contained 82%, 62%, and 52% saponins, respectively [14,15].

**Figure 1:**
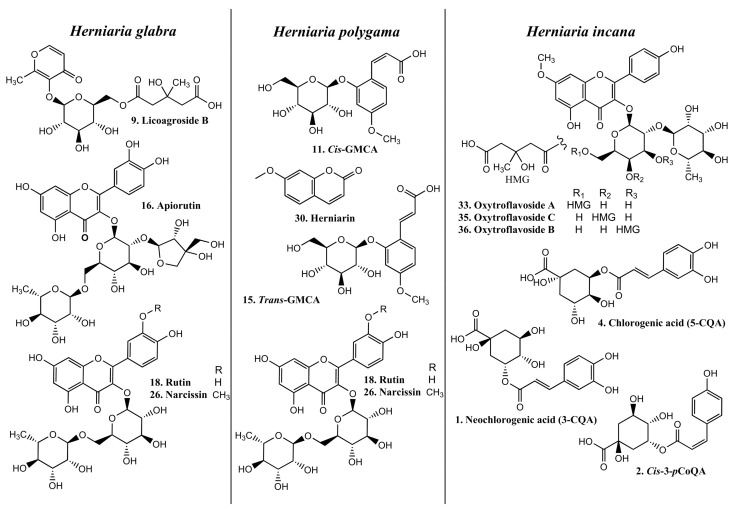
Chemical structures of the main phenolic metabolites in the studied *Herniaria* purified extracts.

**Figure 2:**
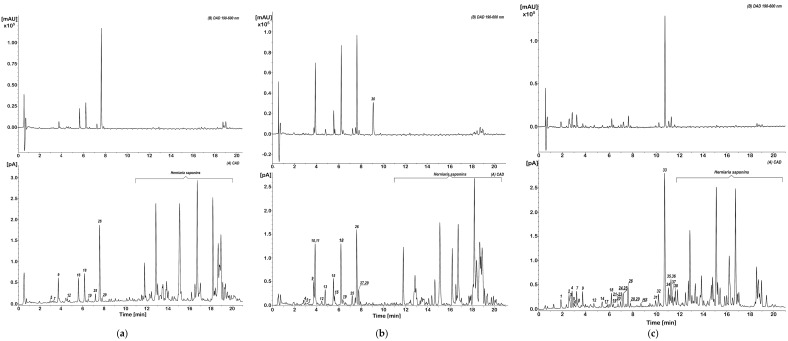
UHPLC—diode array detector (DAD) and charged aerosol detector (CAD) profiles of the purified extracts from (**a**) *Herniaria glabra*; (**b**) *H. polygama*; (**c**) *H. incana* herb.

Commercially available and in-house prepared standards [14,15] were used for the identification and quantification of phenolic compounds in the purified extracts of HG, HP, and HIh. The detailed UV and MS/MS spectra description together with tentative identification of non-triterpenoid constituents in HIh and HP phenolic fractions were presented in our previous work [15]. Due to LC-HRMS analysis 38 *Herniaria*-derived non-saponin compounds were identified—10 in HG-PE, 17 in HP-PE, and 31 in HIh-PE (Figure 1 and Figure 2 and Table 1). The studied objects shared only 9 common HSM, namely *p*-coumaroyl-4-*O*-hexoside (peak 5), *cis*-3-feruloyl quinic acid (FerQA, peak 7), licoagroside B (LB, peak 9), *trans*-4-FerQA (peak 12), rutin (peak 18), quercetin-3-*O*-hexoside (peak 19), nicotiflorin (peak 25), narcissin (peak 26), and isorhamnetin-3-*O*-hexoside (peak 29) (Figure 2). It is worth noting, that both HP and HIh possessed unique fingerprints of phenolics within *Herniaria* species. Namely, HP-PE contained glucosides of 2-hydroxy-4-methoxycinnamic acid (*cis*/*trans*-GMCA, peaks 11 and 15) and 2-hydroxy-4-methoxyphenyl propanoic acid (GMPPA, peak 13), (2*R*,3*R*)-benzoyl-tartaric acid (peak 10), 4,4′-dimethoxy-2,2′-di-*O*-hexoside-truxinate (peak 27). *Cis*/*trans*-GMCA are direct precursors of herniarin (peak 30) and truxinate (peak 27). Both *cis*/*trans* GMCA and herniarin were the main constituents of ethanolic extract from *Chamomilla recutita* L. flowers [18]. Furthermore, *trans*-GMCA, GMPPA as well as 4,4′-dimethoxy-2,2′-di-*O*-hexoside-truxinate were previously identified in *n*-butanol fraction from aerial parts of *Lavandula angustifolia* Mill. [19] HIh-PE contained a variety of 3-hydroxy-3-methylglutaric (HMG) acid conjugates with quercetin (peak 22), 7-*O*-methylquercetin (peak 31), kaempferol (peaks 24, 28), and 7-*O*-methylkaempferol (peaks 33–37). Three non-phenolic metabolites: maltol derivative (LB), *N*-malonyl-tryptophan (peak 14), and a trace amount of blumenin isomer (peak 23) were also identified in these extracts.

Quantification of individual *Herniaria* phenolics, followed by the UHPLC/PDA analysis, was established using the external standard method. The sum of phenolics in HG-PE, HP-PE, and HIh-PE was 12.95%, 24.03%, and 29.56%, respectively. Flavonoids were the predominant group in HG-PE accounting for 11.15%. The major flavonoid constituents included narcissin—7.34% followed by rutin—1.87%, and apiorutin—1.5% (Figure 1, Table 1). Moreover, the contents of narcissin, apiorutin, and LB (1.8%), reported in HG, were the highest within three *Herniaria* spp. The non-terpenoids components of HP-PE comprised of flavonoids—11.69%, cinnamic and benzoic acids derivatives—9.42%, coumarin (herniarin)—1.59%, and LB—1.33%. The significant amounts were documented for *cis*-GMCA—5.76%, narcissin—5.43%, and rutin—5.27% (Figure 1, Table 1). The highest concentration of phenolics was established in HIh. The quantitative profile comprised flavonoids—22.06%, hydroxycinnamic acids (HCA)—6.4%, and LB—1.1%. The most abundant metabolite was oxytroflavoside A accounting for 14.73% (Figure 1, Table 1). This flavonoid is unique among *Herniaria* spp. and was previously revealed only in *Oxytropis falcata* Bunge [20] and *Astragalus gombiformis* Pomel [21] from the family Fabaceae. Moreover, rhamnocitrin, a flavonol aglycone, was first time identified in the family Caryophyllaceae [22]. Among all phenolics in *Herniaria* spp. examined, the highest content was contributed to flavonoids. Detailed phytochemical analysis, carried out in this study, showed that these *Herniaria* taxa should not be used interchangeably due to significant differences in polyphenolic patterns and the content of particular non-saponin constituents.

### 2.2. Evaluation of Antioxidant Effectiveness of the Examined Purified Extracts

**Figure 3:**
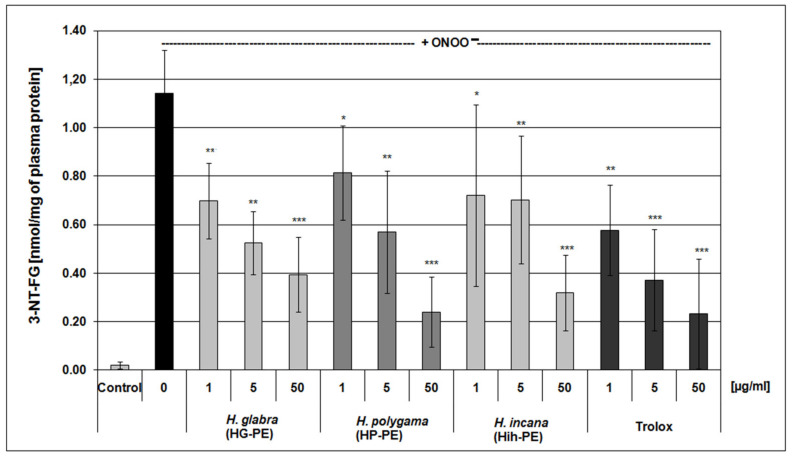
Protective effects of the examined *Herniaria glabra* (HG-PE), *H. polygama* (HP-PE) and *H. incana* herb (HIh-PE) purified extracts on the ONOO^−^-induced formation of 3-nitrotyrosine in blood plasma proteins. Human plasma was exposed to 100 µM ONOO^−^ in the presence or absence of the tested extracts or a reference compound (Trolox); * *p* < 0.05, ** *p* < 0.01, *** *p* < 0.001; *n* = 6.

The exposure of blood plasma to ONOO^−^ action resulted in a significant increase of plasma lipid peroxidation and formation of 3-nitrotyrosine in plasma proteins, accompanied by a decrease of protein thiol groups level. The pre-incubation of blood plasma with the examined *Herniaria* purified extracts noticeably diminished those harmful effects of ONOO^−^ on blood plasma components. The tested PEs (1–50 μg/mL) significantly reduced nitration of tyrosine residues and oxidation of thiol groups in plasma proteins. The anti-nitrative action of the extracts was dose-dependent and comparable to the effects of a well-known reference antioxidant—i.e., Trolox (Figure 3). In measurements of its ferric reducing ability (the FRAP assay), beneficial effects of the *Herniaria* PEs on the non-enzymatic antioxidant capacity were also demonstrated (at concentrations of 5–50 µg/mL). Furthermore, the examined purified extracts displayed moderate anti-lipoperoxidative properties and reduced the TBARS (thiobarbituric acid-reactive substances) level in blood plasma exposed to ONOO^−^, though their actions were not dose-dependent (Table 2).

**Figure 4:**
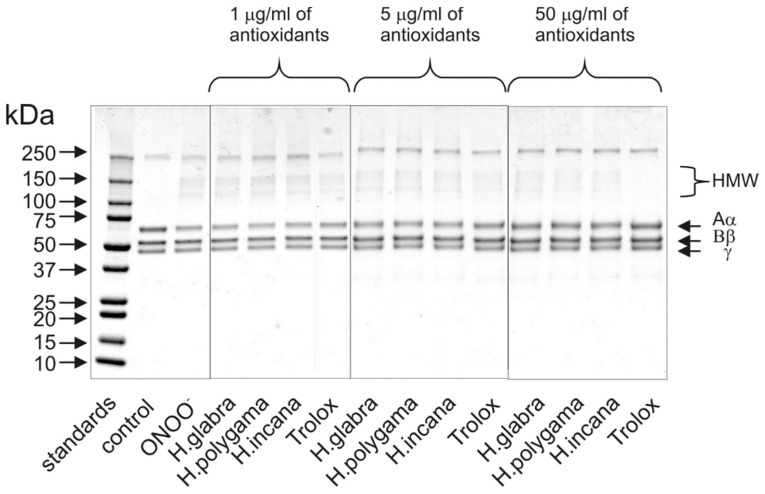
Protective effects of the examined *Herniaria glabra*, *H. polygama* and *H. incana* herb purified extracts on the ONOO^−^-induced oxidative changes in fibrinogen structure. The figure includes a representative SDS-PAGE pattern of human fibrinogen, obtained from the executed experiments employing 4–20% Mini-PROTEAN^®^ TGX™ Precast Protein Gels and staining with Coomassie Brilliant Blue R-250 dye (*n* = 3).

The protective action of the HG-PE, HP-PE, and HIh-PE on blood plasma proteins was additionally confirmed by 1D-electrophoretic analysis (Figure 4). The exposure of fibrinogen to peroxynitrite oxidant resulted in the formation of the high-molecular protein aggregates (HMW), derived from the Aα chain of this protein molecule. Antioxidant effects of all tested extracts were the most evident at a concentration of 50 µg/mL; however, some slight decrease of HMW formation was observed at the concentration of 5 µg/mL (Figure 4).

### 2.3. Anti-Inflammatory Properties of the Tested Herniaria Purified Extracts

**Figure 5:**
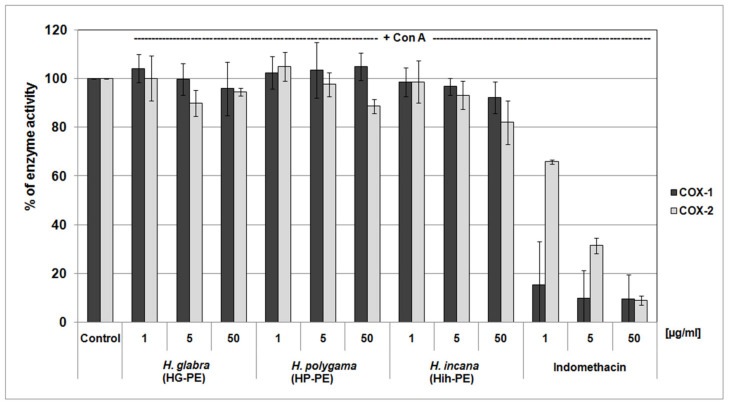
Screening of the cyclooxygenase-inhibitory properties of the examined *Herniaria glabra* (HG-PE), *H. polygama* (HP-PE), and *H. incana* herb (HIh-PE) purified extracts. The enzyme activity in control samples (untreated with the extracts or a reference inhibitor) was assumed as 100%; *n* = 3.

The COX inhibitor screening demonstrated that neither of the examined PEs were able to significantly inhibit the activity of COX-1 nor COX-2. At their highest concentrations (of 50 μg/mL), their inhibitory efficiency mostly did not exceed about 15%. For comparison, the reference anti-inflammatory drug, i.e., indomethacin, effectively inhibited both of the isoenzymes with IC_50_ values of <1 μg/mL (for COX-1) and 2.43 μg/mL (for COX-2) (Figure 5).

**Figure 6:**
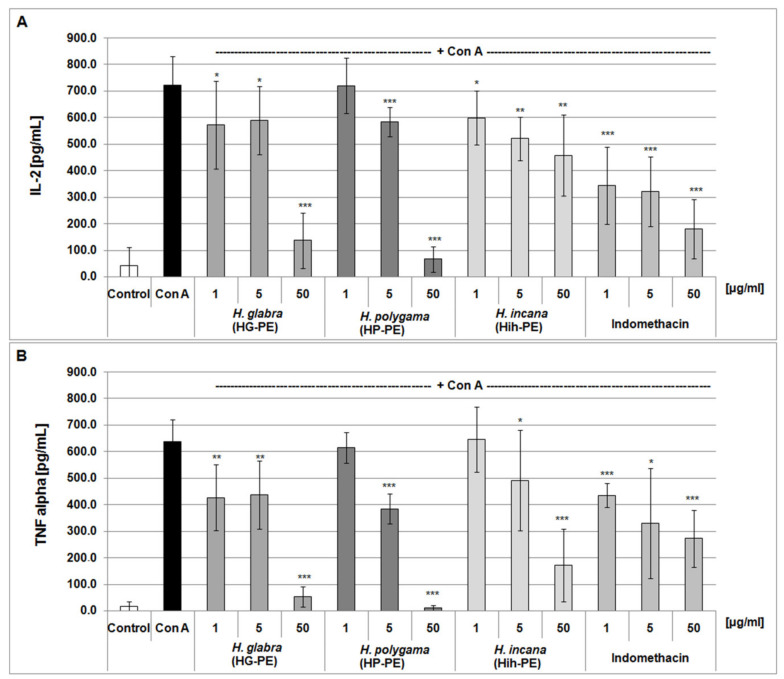
Effects of the examined *Herniaria glabra* (HG-PE), *H. polygama* (HP-PE) and *H. incana* herb (HIh-PE) purified extracts on the inflammatory response of the concanavalin A-stimulated human PBMCs. Anti-inflammatory actions of the tested extracts were estimated using ELISA and based on IL-2 (panel (**A**)) and TNF-α (panel (**B**)) release; * *p* < 0.05, ** *p* < 0.01, *** *p* < 0.001; *n* = 4–5.

However, studies on the concanavalin A (Con A) -stimulated PBMCs clearly indicated that all of the studied *Herniaria* PEs had some anti-inflammatory potential (Figure 6). At the highest concentration of the purified extracts (50 µg/mL), suppression of the inflammatory response of PBMCs reached up to ≥80%, this was estimated by the release of interleukin 2 (IL-2) and tumor necrosis factor alpha (TNF-α). At the lower concentrations of the tested objects (i.e., 1 and 5 µg/mL), their effects were slighter (up to about 20–30% of cytokine level reduction), but mostly also statistically significant.

### 2.4. Cytotoxicity Assay

Preliminary assessment of cytotoxicity was based on a direct exposure of PBMCs (suspended in phosphate-buffered saline) on the examined HG-PE, HP-PE, HIh-PE, without protective conditions of any medium components. Under such experimental conditions, a potential cytotoxic action of the tested purified extracts might be quickly detected (after few hours) [24,25]. The initial viability of control PBMCs in samples was 91 ± 4.5%. Measurements of PBMCs viability revealed no toxic actions of the purified extracts from *H. glabra*, *H. polygama,* and *H. incana* herb at concentrations of 5, 25, and 50 µg/mL (Table 3).

The above observations were additionally verified by the resazurin-based metabolic viability test, conducted after 24 h of incubation of PBMCs with the *Herniaria* purified extracts. No cytotoxic effects were found in the vast majority of the analyzed samples, excluding PBMCs treated with the highest concentration (i.e., 50 µg/mL) of *H. polygama* PE (Table 3).

A significant contribution of enhanced generation of ROS and oxidative stress to etiology and pathophysiology of civilization diseases, including cardiovascular disorders has been well established and widely described [26,27,28,29]. Among natural substances with beneficial effects on human health, ethnomedicinal plants have gained increasing interest as a source of individual bioactive substances, their mixtures, or pharmacophores for the discovery of new drugs [30,31].

Despite the presence in traditional medicine and contemporary herbal therapies, molecular mechanisms and main pathways of biological actions of *Herniaria* species are poorly recognized. So far, only some aspects of the biological actions of *H. glabra* and *H. hirsuta* L. have been described [32,33]. While the most of studies on *H. glabra* are focused on its diuretic [34] and anti-urolithiasis activity [35], other biological actions of different *Herniaria* species remain inadequately recognized. Several reports are describing in vitro anti-oxidant activity of *Herniaria* spp. water-organic and water extracts which were mentioned in our previous paper [15]. As a result, weak (HG, *H. hirsuta* extracts) or moderate (HIh, *H. fontanesii* J. Gay extracts) antioxidant capacity was estimated. However, ethyl acetate fraction from an Egyptian species of *Herniaria* namely *H. hemistemon* J. Gay showed high DPPH^•^ scavenging activity in vitro [36]. It caused 88% scavenging in a dose of 125 µg/mL, this effect was comparable with the ascorbic acid. Flavonoids such as quercetin, naringenin, hesperitin, kaempferol, rhamnetin, apigenin, acacetin and their monoglycosides as well as coumarin were responsible for this activity.

Contrary to those not very promising literature data, our previous study on narcissin, apiorutin, and licoagroside B isolated from *H. glabra* indicated their considerable antioxidant effects [14] and encouraged us to continue work on *Herniaria* plants. Moreover, our recent publication [15] shed new light on the antioxidant activity of *Herniaria*-derived phenolic acid derivatives rich fractions and the 1st one that describes the anti-inflammatory potential of *Herniaria* spp.

Results obtained from the present study indicated that the *Herniaria* genus may not only be a source of natural antioxidants but also covers species displaying anti-inflammatory effects. Antioxidant activity of the examined *Herniaria* purified extracts was evaluated in human blood plasma under the 100 µM peroxynitrite-induced oxidative stress. All of the three examined *Herniaria*-purified extracts (1–50 µg/mL) displayed a considerable antioxidant action in blood plasma. The use of peroxynitrite (ONOO^−^) as an inductor of oxidative stress was based on the pathological significance of this ROS. It is one of the main oxidants generated within the cardiovascular system, responsible for both nitrative and oxidative damage to blood cells and blood plasma components [37,38,39,40,41,42]. For that reason, natural substances capable of detoxifying (scavenging) ONOO^−^ may be crucial for the prevention of oxidative stress and its functional consequences. The literature data indicate that the bolus addition of 250 µM ONOO^−^ to the experimental system reflects a physiological concentration of 1 µM ONOO^−^, maintained for 7 min [43], which is locally achievable at sites of inflammation. Our assays revealed that all of the tested objects diminished the ONOO^−^-induced plasma lipid peroxidation as well as the formation of 3-nitrotyrosine and oxidation of thiol groups in blood plasma proteins (Table 2 and Figure 3). In vivo, the maintaining of the physiological antioxidant capacity of blood plasma is crucial for intravascular homeostasis; therefore, in this study, the *Herniaria* PEs effects on this parameter were also determined. For the first time, we have demonstrated that the examined preparations may protect or even strengthen the physiological non-enzymatic antioxidant capacity of blood plasma. Experiments on the isolated fibrinogen demonstrated that the presence of the *Herniaria* purified extracts (before the exposure to ONOO^−^) partly reduced the formation of HMW, derived from the Aα chain of this molecule (Figure 4). The ability of the examined plant preparations to protect fibrinogen, one of the key proteins in the blood coagulation cascade, is very important from the physiological point of view. Due to its spread structure and physiological exposure of protein surface to other molecules, fibrinogen is very prone to undesirable modifications, including oxidative damage. Both oxidative and nitrative alterations in fibrinogen structure may lead to functional consequences, including changes in its clotting ability and interactions with blood platelets [44,45,46]. For instance, it has been shown that even very low nitration of fibrinogen molecule (≈45–65 μmol nitrotyrosine/mol tyrosine) may significantly accelerate the formation of a fibrin clot when compared to native fibrin(ogen) [44]. Moreover, a recent systematic analysis has also confirmed that post-translational modifications of fibrinogen affect both the fibrin clot formation and its structure [47]. Peroxynitrite is also able to weaken functions of the fibrinolytic proteins [48,49]. It should be also emphasized that the HG-PE, HP-PE, HIh-PE displayed significant antioxidant efficacies at concentrations of 1–5 µg/mL, which are comparable to physiologically achievable levels of plant-derived substances or their metabolites in blood plasma, i.e., ≤5–10 µM [50]. Antioxidant effects of the investigated *Herniaria* purified extracts may be attributed to the presence of numerous polyphenolic compounds belonging to different groups of phytochemicals (Figure 1 and Figure 2, Table 1). Of course, the best known of them is rutin (quercetin-3-*O*-rutinoside), possessing a wide range of beneficial physiological effects, including the ability to prevent or ameliorate oxidative stress [51,52,53]. However, according to our previous studies, also narcissin, apiorutin and licoagroside B have antioxidant properties and might prevent oxidative and nitrative damage caused by peroxynitrite [14].

Moreover, our studies demonstrated the anti-inflammatory potential of the examined *Herniaria* purified extracts. It was in line with our previous results on the anti-inflammatory activity of phenolic acid derivatives rich fractions from HP and HIh [15]. Our preliminary screening involving the exposure of purified COXs (a commercial kit) to the *Herniaria* PEs did not show the direct inhibitory action of these plant preparations towards the COX-2 enzyme (Figure 5). However, experiments on PBMC cultures showed that their inflammatory response was significantly reduced by all purified *Herniaria* extracts. Thus, it is likely that the examined phytochemicals may upregulate the activation of pro-inflammatory pathways and act rather as regulators of gene expression than direct inhibitors of pro-inflammatory enzymes. At their physiologically relevant concentrations (1–5 µg/mL), the tested plant preparations reduced the inflammatory response of PBMCs, leading to about a 20% and 30% decrease of IL-2 and TNF-α secretion, respectively (Figure 6). Additionally, cytotoxicity tests confirmed the cellular safety of all tested extracts at these concentrations (Table 3). The purified extract from *H. glabra* at the lower doses proved to be the most efficient suppressor of PBMCs’ inflammatory response. HG-PE at 1 µg/mL inhibited TNF-α release comparably to the reference compound—indomethacin at the same dose. In contrast, *H. polygama* purified extract at a dose of 50 µg/mL (the highest dose tested), was the most active among the tested preparations, including positive control. Nevertheless, this dose exceeded the physiologically relevant concentrations and was cytotoxic against PBMCs in the resazurin-based assay. The described effects can be explained by the presence of high amounts of rutin (2.8 times as much in HP-PE as in HG-PE) and narcissin (1.3 times as much in HG-PE as in HP-PE) in both extracts, while *H. glabra* preparation contains the highest amount of saponins. On the other hand, *H. polygama* purified extract composed of rare HCA conjugates, namely *cis*-GMCA (5.76%), *tran*s-GMCA (1.58%), GMPPA (1.07%) and coumarin derivative—herniarin (1.59%). These metabolites were also isolated from plants with recognized anti-inflammatory properties, i.e., *Chamomilla recutita*, *Lavandula angustifolia*, and *Artemisia dracunculus* L. (Tarragon) [54]. Although the examined HP-PE showed higher inhibitory activity against IL-2 and TNF-α than the fraction of phenolic acid derivatives from *H. polygama* (consisting of *cis*-GMCA 29.7%, GMPPA 5.2%, *trans*-GMCA 7.59%, herniarin 0.82%, benzoyl tartaric acid 5.5%) [15]. It may be explained by the synergic and additive effects of the main groups of HSM, i.e., saponins, flavonoids, phenolic acids, and coumarin. The synergistic anti-inflammatory effect of flavonoids mixtures [55] and additive antioxidant action of flavonoids and cinnamic acid mixes have been described [56].

The obtained results suggest the ability of the *Herniaria* PEs-derived metabolites to suppress the activation of pro-inflammatory pathways and are consistent with existing literature data. The pro-inflammatory response of cells involves various signal transduction and metabolic pathways. Thus, inflammation can be diminished at diverse molecular levels, including by direct action on the active COX-2 enzyme or other pro-inflammatory enzymes as well as by modulation of gene expression (i.e., reducing pro-inflammatory genes transcription). One of the key molecular regulators of the cellular pro-inflammatory response is the activation of the nuclear factor kappa-light-chain-enhancer of activated B cells (NF-κB) [57]. The literature indicates that prevention of NF-κB activation is one of the most important mechanisms of anti-inflammatory action of various phytochemicals. For instance, rutin (which is also one of the components of the examined *Herniaria* extracts, and in the highest content (5.27%) detected in *H. polygama*) was found to inhibit NF-κB induction. Blockage of NF-κB activation results in the inhibition of other signaling pathways, dependent on this transcription factor, including cytokine release, pro-inflammatory enzymes synthesis and expression of other molecules involved in the inflammatory response [58]. The literature also provides data on the NF-κB inhibitory effect of other metabolites found in the studied PEs, i.e., chlorogenic [59] and neochlorogenic acids [60].

The recent evidence highlighted the molecular mechanism of anti-inflammatory action of Samoan traditional medicine (*Psychotria insularum* homogenate) that was mediated via iron chelation [61]. Iron chelation is also involved in antioxidant and antibacterial effects. The bioactive principle of the *P. insularum* homogenate was based on the reduction of the intracellular iron content as well as heme synthesis in yeast (*Saccharomyces cerevisiae*) cells. Bioassay-guided fractionation of the *Psychotria insularum* homogenate discovered of bioactive iron chelators namely rutin and nicotiflorin. Rutin was more active due to the presence of the catechol group in its structure. Immune response assay of the homogenate as well as bioactive constituent rutin, and the reference drug—ibuprofen, was tested on unstimulated, ConA-stimulated, or LPS-stimulated splenocytes isolated from C57BL/6 mice. Overall, fever-inducing cytokine TNFα and Th1 (T helper cells type 1) -inducing IL12p40 together with proinflammatory cytokines Th1 (interferon γ) and Th17 (IL6, IL17A) were significantly reduced by the tested objects. The anti-inflammatory activity of rutin and the homogenate was consistent with ibuprofen. The mention flavonoids were also detected in the studied *Herniaria* PE, rutin amount was higher, than nicotiflorin (Table 1).

Most of the available evidence concerns the anti-inflammatory action of chlorogenic acid [62]. For instance, in the lipopolysaccharide-stimulated RAW 264.7 cells, chlorogenic acid was found to inhibit the expression of COX-2 and the inducible nitric oxide synthase (iNOS), and its anti-inflammatory properties were associated with inhibitory action on NF-κB [63].

The capability of inhibiting NF-κB was also described for kaempferol [64] and some of its derivatives [65]. However, the number of papers dealing with activities of other main components of the examined purified extracts, e.g., narcissin, *cis*-GMCA, and oxytroflavoside A are limited. Besides our previous work on antioxidant properties of narcissin [14], the biological properties of this compound have been described in a few papers. Some data on antimicrobial and immunomodulatory activities of narcissin were provided by [66]. According to these authors, this compound reduced the lipopolysaccharide-induced release of IL-1β and IL-6 from human macrophages. 

The anti-inflammatory potential of the *Herniaria*-purified extracts may also be explained by the presence of triterpenoid saponins. Herniariasaponins are represented by sapogenins of medicagenic and zanhic acids [2]. There is a limited number of scientific reports that have described the anti-inflammatory potential of the mentioned saponins. Chen et al. studied the medicagenic acid-3-*O*-β-D-glucopyranoside (at doses 10, 20, 40 mg/kg) isolated from *Dolichos falcata* Klein on the models of the monosodium urate crystals-treated monocyte/macrophage cell line RAW 264.7 in vitro for its possible mechanism on gout arthritis [67]. Anti-inflammatory effect of the studied compound manifested via strong suppression of the pro-inflammatory cytokines production level of TNF-α, IL-1β, and IL-6 in the dose-dependent manners. Another study by Vo et al. revealed the 5-LOX inhibitory activity of medicagenic acid (IC_50_ = 30.4 μM) in vitro [68]. Nevertheless, it did not inhibit COX-1 and very low COX-2 isoenzymes, which is consistent with our results.

## 3. Materials and Methods

### 3.1. Chemicals and Biological Material

Acetonitrile, methanol (HPLC and LC-MS grade), formic acid (98–100% purity and MS-grade) were purchased from Merck (Darmstadt, Germany). 5-CQA (Aldrich, ≥95%), 4-CQA (Sigma, ≥95%), and herniarin (Roth, 98%) were commercial standards, licoagroside B, apiorutin, rutin, and narcissin were previously isolated from *H. glabra* L. [14]. Kaempferol was in-house prepared at the Department of Biochemistry and Crop Quality, IUNG. Ultrapure water was obtained in-house with a purification system (Milli-Q^®^ Simplicity 185, Millipore Corp., Billerica, MA, USA).

Trichloroacetic and thiobarbituric acids, dimethyl sulfoxide (DMSO), phosphate-buffered saline (PBS), tris, Trolox, Sigma Fast OPD substrate for peroxidase, and the resazurin-based in vitro toxicology assay kit were purchased from Sigma-Aldrich (St. Louis, MO, USA). Immunochemical reagents for the enzyme-linked immunosorbent assay (ELISA) such as primary anti-3-nitrotyrosine antibody, biotinylated secondary antibody, and Streptavidin/HRP complex were from Abcam (Cambridge, UK). Peroxynitrite was synthesized according to a method described by Pryor et al. (1995) [69]. BCA Protein Assay Kit was purchased from ThermoFisher Scientific (Waltham, MA, USA). 4–20% Mini-PROTEAN^®^ TGX™ Precast Protein Gels and other reagents for SDS-PAGE were provided by BioRad Laboratories (Hercules, CA, USA). COX Colorimetric Inhibitor Screening Assay Kit (item No. 701050) was purchased from Cayman Chemicals (Ann Arbor, MI, USA). Determination of human interleukin-2 (IL-2) and tumor necrosis factor-alpha (TNF-α) was conducted with the use of Picokine^®^ ELISA kits (items No. EK0397 and EK0525, respectively) were purchased from BosterBio (Pleasanton, CA, USA). Other analytical reagents were purchased from local or international suppliers.

Antioxidant and anti-inflammatory assays were executed based on commercially available blood units (buffy coats), purchased from the Regional Centre of Blood Donation and Blood Treatment in Lodz, Poland. This material was used to isolate blood plasma, fibrinogen, and peripheral blood mononuclear cells (PBMCs). Fibrinogen isolation from blood plasma was carried out with the cold ethanol precipitation technique [70]. Stock solutions of the examined extracts were prepared with 25% DMSO, providing its final concentration in the assayed samples of ≤0.025%.

### 3.2. Plant Material

The studied plant materials were wild-grown in Ukraine and collected during the flowering stage. The entire herb of *Herniaria glabra* L. was harvested in July 2016, Ternopil Oblast (GPS-Coordinates: 49°45′23″ N, 25°30′38″ E). The whole plant of *H. polygama* J. Gay was collected in July 2016, Kyiv Oblast (GPS-Coordinates: 50°26′48.8′′ N, 30°02′38.7′′ E). The mentioned plant objects were harvested by Solomiia Kozachok and identified by Dr. Liudmyla Zavialova (Dept. of Geobotany and Ecology, M.G. Kholodny Inst. of Botany, NAS of Ukraine, Kyiv, Ukraine). The areal parts of *H. incana* Lam. wild growing in Odesa Oblast (Mayaky village near the Astronomical Observatory) were collected in May 2017 and identified by Dr. Olena Bondarenko (Dept. Botany, Odesa I.I. Mechnikov National Univ., Odesa, Ukraine).

Harvested plant materials were naturally dried in a well-ventilated space with no direct sunlight under the gauze. Following, the dried materials were ground using an Ultra Centrifugal Mill ZM 200 (Retsch, Germany) with 0.5 mm sieve pores. The plant’s powder was transferred into tightly closed containers and kept in a dark cold place until the analysis.

### 3.3. Extraction

The preparation method of the *Herniaria* purified extracts was described in detail in our previous work [15]. Briefly, extraction was performed using ASE 200 (accelerated solvent extraction) Dionex system with 80% methanol-water solution. Following the obtained extracts were defatted with *n*-hexane, evaporated up to water phase using a rotary evaporator (Heidolph, Schwabach, Germany), and applied to preconditioned RP-C18 open column (50 mm × 93 mm i.d., Cosmosil 140C18-PREP, 140 μm). HSM were rinsed with 95% methanol solution, evaporated and finally lyophilized using Gamma 2–16 LSC freeze dryer (Martin Christ Gefriertrocknungsanlagen GmbH, Germany). The obtained dried powders were the studied research objects, namely HG-PE, HP-PE, and HIh-PE.

### 3.4. LC-MS Analysis

The purified *Herniaria* extracts were subjected to LC-MS analysis. The analysis for tentative identification of HSM was run on the Thermo Scientific Ultimate 3000 RS (Thermo Fischer Scientific, Waltham, MS, USA) chromatographic system hyphenated to Bruker Impact II HD (Bruker, Billerica, MA, USA) QTOF-MS (quadrupole-time of flight mass spectrometer), and both a CAD (Thermo Corona Veo RS) and PDA detectors. The separations were performed on an ACQUITY HSS T3 column (100 × 2.1 mm i.d.; 1.8 μm, Waters Corp., Milford, CT, USA) at 60 °C. The settings of MS and chromatographic method were described in detail in our previous publication [15].

Quantity analysis of the phenolic metabolites in HG-PE, HP-PE, HIh-PE was carried out in triplicate using a Waters ACQUITY UPLC system (Waters Corp., Milford, MA, USA) equipped with a binary pump, and TQD-MS (tandem triple-quadrupole mass spectrometer) and DAD detectors as was recorded earlier [15]. The purified *Herniaria* extracts were dissolved in 70% MeOH (*v*/*v*) at a concentration of 10 mg/mL, centrifuged at 16,000× *g* (Polygen Sigma 3-16KL, D-37520 Osterode am Harz, Germany), and then subjected to analysis. The amount of individual non-saponin compounds were calculated by the external standard method. Calibration curves for (2*R*,3*R*) benzoyl-tartaric acid, 5-CQA, narcissin, herniarin, and licoagroside B were prepared in six concentrations (5, 50, 100, 150, and 250 μg/mL) and measured at UV 275, 320, 350, 323, and 257 nm respectively. 5-CQA was a group standard for cinnamic acid derivatives, (2*R*,3*R*) benzoyl-tartaric acid—for GMPPA, and narcissin—for flavonoids. Molar absorption coefficients (ε) of *trans*-GMCA, *cis*-GMCA, GMPPA, 7-*O*-methylquercetin-3-*O*-[HMG-(1→6)]-[α-Rha-(1→2)]-β-galactoside, oxytroflavoside A, rutin, kaempferol, and apiorutin (1845 m^2^/mol at 350 nm), as well as group standards, were calculated [15]. Their values were used for the correction of chromatographic peaks areas to quantify compounds for which calibration curves were absent.

### 3.5. Evaluation of Antioxidant Effects in Blood Plasma

Plasma was pre-incubated for 15 min at 37 °C with the crude extracts (1–50 μg/mL) or a reference compound, i.e., Trolox (1–50 μg/mL), and then underwent the exposure to ONOO^−^. For the ferric reducing ability of blood plasma (FRAP) assay, the final concentration of ONOO^−^ was 150 μM, while in other antioxidant assays it was 100 μM. Plasma treated with ONOO^−^ in the absence of the purified extracts or Trolox was also prepared. Control plasma contained neither the examined substances nor ONOO^−^.

Evaluation of antioxidant actions of the *Herniaria* purified extracts in blood plasma covered measurements of thiobarbituric acid-reactive substances (TBARS, 3-nitrotyrosine, and protein thiol groups levels. TBARS concentration was determined according to the method described previously by Wachowicz and Kustron (1992) [71]. For the immunodetection of 3-nitrotyrosine, a competitive ELISA was applied [72]. All results from this assay were expressed as equivalents of a 3-nitrotyrosine-containing protein standard (i.e., nitrated fibrinogen, 3-NT-FG) per mg of blood plasma protein. Thiol groups in blood plasma protein were determined using Ellman’s reagent [73].

The purified extracts influence of the non-enzymatic antioxidant capacity (NEAC) of blood plasma was measured in the FRAP assay, based on the determination of the capability of the analyzed samples of reducing the ferric ions (Fe^3+^) to ferrous ions (Fe^2+^). Plasma samples were assayed according to our previous procedure; the results were calculated from a standard curve and expressed as Fe^2+^ equivalents [74].

### 3.6. Evaluation of Anti-Inflammatory Properties

Cyclooxygenase (COX-1 and -2) enzymes inhibitor screening of the examined *Herniaria* purified extracts (1–50 µg/mL) and a reference inhibitor (i.e., indomethacin, 1–50 µg/mL) was executed using a commercial colorimetric kit, based on the oxidation of *N,N,N’,N’*-tetramethyl-*p*-phenylenediamine (TMPD) chromogenic substrate. The method allowed to measure the enzymatic activity of the peroxidase component of COX. Assays were carried out in triplicate. 

Anti-inflammatory properties of the HG-PE, HP-PE, and HIh-PE were also determined employing PBMCs under the Con A-induced stimulation. The cells were cultured in RPMI 1640 medium (supplemented with 10% fetal calf serum and 0.1% of penicillin-streptomycin), at a density of 1.5 × 10^6^ cells/mL. The 1h-preincubation of PBMCs with the examined purified extracts in a laboratory CO_2_ incubator was followed by treatment with Con A (at the final conc. of 10 µg/mL), to induce their pro-inflammatory activity. Then, PBMCs were cultured for 24 hrs (in 96-well microplates, at 37 °C, with 5% of CO_2_ concentration and 95% humidity). After 24 hrs, microplates were centrifuged to obtain supernatants (cell culture medium) for further analyses, involving immune-enzymatic determinations of interleukin-2 and TNF-α release from the cells. Concentrations of the cytokines were established according to protocols, provided by the manufacturer.

### 3.7. Cytotoxicity Assays

Preliminary evaluation of cellular safety of the examined extracts was based on short-term incubation cytotoxicity tests [24,25]. PBMCs, suspended only in 0.02 PBS (1 × 10^6^ PBMCs/mL) were incubated with the extracts (5, 25, and 50 µg/mL) to directly expose the cells to the examined *Herniaria* preparations and assess the possibility of direct damage to the cell membrane. Cytotoxicity was measured after 6 h of incubation (at 37 °C, with gentle mixing on a rotary shaker) in a micro-chamber automated cell counter, employing the trypan blue excluding test [75]. Preliminary data on cellular safety of the extracts, derived from trypan blue assays, were additionally confirmed in the resazurin-based cell viability assay. For this assay, PBMCs (1.5 × 10^6^ PBMCs/mL) were suspended in the RPMI-1640 medium (supplemented with 10% fetal calf serum and 0.1% of penicillin-streptomycin) and incubated with the *Herniaria* purified extracts (5, 25 and 50 μg/mL) for 24 h (in 96-well microplates, at 37 °C, with 5% of CO_2_ concentration and 95% humidity). After the incubation, the resazurin-based viability tests were carried out. PBMCs viability was determined using a microplate spectrophotometer BMG Labtech SectroStarNano, at λ = 600 nm (690 nm was used as a reference wavelength).

### 3.8. Determination of Changes in Fibrinogen Structure by 1D-Electrophoresis

Analogously to blood plasma samples, human fibrinogen (2 mg/mL, in 0.02 M PBS) was pre-incubated with the tested *Herniaria* purified extracts or Trolox, and then, exposed to ONOO^−^ (100 μM). The SDS-PAGE [76] separations were executed under reducing conditions, using 4–20% Mini-PROTEAN^®^ TGX™ Precast Protein Gels and BioRad MiniProtean Tetra Cell equipment for 1-D vertical gel electrophoresis. Protein bands were visualized using Coomassie Brilliant Blue R-250 dye.

### 3.9. Statistical Analysis

All the values in this work are expressed as mean ± SD; *p* < 0.05 was assumed as statistically significant; *n* = number of blood donors. In all experiments on blood plasma and PBMCs, at least two independent incubations of the examined substances with biological material from each donor were performed. Statistical significance of the obtained results was evaluated using the Student’s t-test or ANOVA and Tukey’s post hoc test.

## 4. Conclusions

The performed results describe the composition and concentration levels of single non-saponin constituents in the purified extracts of *Herniaria glabra*, *H. polygama*, and *H. incana* herb. The phenolic metabolites were dominated by narcissin (7.34%) in HG-PE, *cis*-2-hydroxy-4-methoxycinnamic acid 2-*O*-β-glucopyranoside (5.76%), narcissin (5.43%), and rutin (5.27%) in HP-PE, and oxytroflavoside A (14.73%) in HIh-PE. The *Herniaria* purified extracts exhibited considerable anti-oxidant activities in blood plasma under the peroxynitrite-induced oxidative stress in a dose of 1–5 µg/mL. Moreover, the *Herniaria*-derived preparations partly reduced the formation of the high-molecular aggregates, derived from the Aα chain of this molecule. The studied objects reduced pro-inflammatory response in the peripheral blood mononuclear cells induced with concanavalin A by the suppressive effect on the level of TNF-α, IL-2. The most efficient suppressor of PBMCs response was the purified extract from *H. glabra*. No toxicity towards PBMCs was revealed for the studied *Herniaria* purified extracts in vitro. 

Our results shed new light on phenolic profiles of rupturewort taxa and revealed unique phenolic markers to distinguish *H. polygama* and *H. incana* from other *Herniaria* spp. Moreover, the in vitro bioassay data indicated the anti-oxidant and anti-inflammatory potential of the studied rupturewort species. Based on the obtained results, we intend to examine in detail the saponin profiles of all three *Herniaria* taxa and revise the view of whether *H. glabra* can be used interchangeably with *H. incana* as suggested by the EMA monograph and *H. polygama* herb as proposed by Ethnobotanical evidence.

## Figures and Tables

**Table 1:** Non-saponin compounds identified and quantified in *Herniaria glabra* (HG), *H. polygama* (HP), *H. incana* herb (HIh) purified extracts using UHPLC-QTOF-MS/MS.

	t_R_ (min)	Compound	Neutral Formula	Mode	m/z	Δ *	Mσ **	Major Fragments (%)	HG,%	HP,%	HIh,%	Ref
1	1.89	3-CQA (Neochlorogenic acid)	C_16_H_18_O_9_	[M − H]^−^ [2M − H]^−^	353.0872 707.1820	1.6 0.9	1.5 1.3	191.0558 (100) (C_7_H_11_O_6_), 179.0342 (80.5) (C_9_H_7_O_4_), 173.0342 (3.5) (C_7_H_9_O_5_), 161.0236 (4.8) (C_9_H_5_O_3_), 135.0439 (14.1) (C_8_H_7_O_2_)	_	_	1.27	[15]
2	2.56	*Cis*-3-*p*CoQA	C_16_H_18_O_8_	[M − H]^−^ [2M − H]^−^	337.0923 675.1926	1.6 0.74	6.8 9.3	191.0559 (7.1) (C_7_H_11_O_6_), 173.0453 (4.1) (C_7_H_9_O_5_), 163.0390 (100) (C_9_H_7_O_3_), 155.0336 (2.3) (C_7_H_7_O_4_), 119.0487 (8.6) (C_8_H_7_O)	_	_	1.51	[15]
3	2.64	*Trans*-3-*p*CoQA	C_16_H_18_O_8_	[M − H]^−^ [2M − H]^−^	337.0921 675.1919	2.5 1.8	4.3 8.6	191.0558 (37.6) (C_7_H_11_O_6_), 173.0449 (4.4) (C_7_H_9_O_5_), 163.0392 (100) (C_9_H_7_O_3_), 155.0343 (2.0) (C_7_H_7_O_4_), 119.0489 (9.2) (C_8_H_7_O)	_	_	0.31	[15]
4	2.81	^a^ 5-CQA (Chlorogenic acid)	C_16_H_18_O_9_	[M − H]^−^ [2M − H]^−^	353.0871 707.1822	0.7 1.0	1.9 23.5	191.0556 (100) (C_7_H_11_O_6_), 161.0244 (1.53) (C_9_H_5_O_3_)	_	TA	1.37	[15]
5	2.99	*p*CoA-4-*O*-hexoside	C_15_H_18_O_8_	[M − H]^−^ [2M − H]^−^	325.0923 651.1925	1.8 0.8	9.7 11.4	163.0393 (100) (C_9_H_7_O_3_), 119.0490 (12.6) (C_8_H_7_O)	TA	TA	0.12	[15]
6	3.05	^a^ 4-CQA (Cryptochlorogenic acid)	C_16_H_18_O_9_	[M − H]^−^ [2M − H]^−^	353.0874 707.1829	1.2 −0.1	3.6 15.3	191.0552 (40.4) (C_7_H_11_O_6_), 179.0343 (84.5) (C_9_H_7_O_4_), 173.0446 (100) (C_7_H_9_O_5_), 161.0231 (2.7) (C_9_H_5_O_3_), 155.0330 (5.3) (C_7_H_7_O_4_), 135.0441 (13.9) (C_8_H_7_O_2_)	_	_	0.20	[15]
7	3.21	*Cis*-3-FerQA	C_17_H_20_O_9_	[M − H]^−^ [2M − H]^−^	367.1025 735.2128	2.6 1.9	4.0 15.7	193.0500 (100) (C_10_H_9_O_4_), 173.0450 (4.0) (C_7_H_9_O_5_), 155.0343 (1.2) (C_7_H_7_O_4_), 149.0597 (2.0) (C_9_H_9_O_2_), 134.0363 (C_8_H_6_O_2_^●^) (6.1)	TA	TA	1.24	[15]
8	3.31	*Trans*-3-FerQA	C_17_H_20_O_9_	[M − H]^−^ [2M − H]^−^	367.1030 735.2138	1.2 0.6	6.7 36.2	193.0502 (100) (C_10_H_9_O_4_), 173.0445 (5.5) (C_7_H_9_O_5_), 155.0338 (1.0) (C_7_H_7_O_4_), 149.0599 (4.3) (C_9_H_9_O_2_), 134.0365 (11.8) (C_8_H_6_O_2_^●^)	_	_	0.16	[15]
9	3.72	^#^ Licoagroside B (maltol-3-*O*-[HMG-(1→6)]-β-glucoside)	C_18_H_24_O_12_	[M − H]^−^ [2M − H]^−^	431.1189 863.2463	1.3 0.9	4.3 7.2	161.0448 (10) (C_6_H_9_O_5_), 125.0232 (100) (C_6_H_5_O_3_)	1.80	1.33	1.10	[14]
10	3.81	^#^ (2*R*,3*R*)-Benzoyl-tartaric acid	C_11_H_10_O_7_	[M − H]^−^	253.0340	5.4	6.8	130.9969 (26.3) (C_4_H_3_O_5)_, 121.0277 (100) (C_7_H_5_O_2_)	_	1.01	_	[15]
11	3.87	^#^*Cis*-2-hydroxy-4-methoxycinnamic acid 2-*O*-β-glucoside (*cis*-GMCA)	C_16_H_20_O_9_	[M − H]^−^	355.1017	5.2	4.9	193.0494 (100) (C_10_H_9_O_4_), 149.0591 (75.4) (C_9_H_9_O_2_), 134.0367 (5.5) (C_8_H_6_O_2_^●^)	_	5.76	_	[15]
12	4.70	*Trans*-4-FerQA	C_17_H_20_O_9_	[M − H]^−^	367.1028	1.9	4.1	193.0501 (21.3) (C_10_H_9_O_4_), 191.0558 (40.6) (C_7_H_11_O_6_), 173.0455 (100) (C_7_H_9_O_5_), 155.0349 (4.0) (C_7_H_7_O_4_), 134.0372 (2.5) (C_8_H_6_O_2_^●^)	TA	TA	0.22	[15]
13	4.77	^#^ 3-(2-glucosyloxy-4- methoxyphenyl)propanoic acid (GMPPA)	C_16_H_22_O_9_	[M − H]^−^	357.1174	4.9	0.7	195.0650 (100) (C_10_H_11_O_4_), 177.0541 (2.8) (C_10_H_9_O_3_), 151.0748 (10.7) (C_9_H_11_O_2_), 136.0512 (1.1) (C_8_H_8_O_2_^●^)	_	1.07	_	[15]
14	5.41	*N*-Malonyl-tryptophan	C_14_H_14_N_2_O_5_	[M + H]^+^	291.0969	2.3	4.6	273.0868 (19.0) (C_14_H_13_N_2_O_4_), 245.0916 (100) (C_13_H_13_N_2_O_3_), 227.0807 (24.9) (C_13_H_11_N_2_O_2_), 188.0702 (21.9) (C_11_H_10_NO_2_), 130.0647 (18.7) (C_9_H_8_N)	_	_	NC	[15]
15	5.52	^#^*Trans*-2-hydroxy-4- methoxycinnamic acid 2-*O*-β-glucoside (*trans*-GMCA)	C_16_H_20_O_9_	[M − H]^−^	355.1016	5.3	0.7	193.0494 (100) (C_10_H_9_O_4_), 149.0591 (36.5) (C_9_H_9_O_2_), 134.0357 (4.7) (C_8_H_6_O_2_^●^)	_	1.58	_	[15]
16	5.60	^#^ Apiorutin (quercetin-3-*O*-[β-Api-(1→2)]-[α-Rha-(1→6)]-β-glucoside)	C_32_H_38_O_20_	[M − H]^−^	741.1868	2.1	5.9	609.1461 (0.5) (C_27_H_29_O_16_), 591.1362 (0.3) (C_27_H_27_O_15_), 300.0269 (100) (C_15_H_8_O_7_^●^), 271.0244 (4.5) (C_14_H_7_O_6_), 255.0299 (2.0) (C_14_H_7_O_5_), 178.9986 (2.0) (C_8_H_3_O_5_), 151.0028 (0.9) (C_7_H_3_O_4_)	1.50	0.33	_	[14]
17	5.83	Kaempferol-3-*O*-(dHex-(1→2))-[dHex-(1→2)]- hexoside	C_33_H_40_O_19_	[M − H]^−^	739.2080	1.5	29.0	575.1392 (0.6) (C_27_H_27_O_14_), 284.0316 (100) (C_15_H_8_O_6_^●^), 255.0291 (7.4) (C_14_H_7_O_5_), 227.0341 (4.5) (C_13_H_7_O_4_), 178.9973 (1.2) (C_8_H_3_O_5_), 151.0024 (1.5) (C_7_H_3_O_4_)	_	_	0.16	[15]
18	6.18	^#^ Rutin (quercetin-3-*O*-[α-Rha-(1→6)]-β-glucoside)	C_27_H_30_O_16_	[M − H]^−^	609.1453	1.5	8.3	343.0459 (1.7) (C_17_H_11_O_8_), 300.0268 (100) (C_15_H_8_O_7_^●^)_,_ 255.0299 (1.0) (C_14_H_7_O_5_), 178.9986 (2.9) (C_8_H_3_O_5_), 151.0037 (1.8) (C_7_H_3_O_4_)	1.87	5.27	0.82	[14]
19	6.33	Quercetin-3-*O*- hexoside	C_21_H_20_O_12_	[M − H]^−^	463.0874	1.6	4.8	343.0459 (1.4) (C_17_H_11_O_8_), 300.0267 (100) (C_15_H_8_O_7_^●^), 255.0299 (1.1) (C_14_H_7_O_5_), 178.9965 (1.8) (C_8_H_3_O_5_), 151.0035 (1.5) (C_7_H_3_O_4_)	TA	0.22	0.18	
20	6.76	Kaempferol-3-*O*-[dHex-(1→6)]- hexoside	C_27_H_30_O_15_	[M − H]^−^	593.1499	1.3	2.1	327.0502 (3.2) (C_17_H_11_O_7_), 285.0389 (53.9) (C_15_H_9_O_6_), 284.0317 (100) (C_15_H_8_O_6_^●^), 255.0300 (2.9) (C_14_H_7_O_5_), 227.0343 (1.7) (C_13_H_7_O_4_), 151.0035 (2.1) (C_7_H_3_O_4_)	_	_	0.19	[15]
21	6.95	Kaempferol-3-*O*-hexoside	C_21_H_20_O_11_	[M − H]^−^	447.0902	2.0	6.5	284.0322 (47.4) (C_15_H_8_O_6_^●^)	_	_	TA	[15]
22	6.96	Quercetin- 3-*O*-[HMG]- hexoside	C_27_H_28_O_16_	[M − H]^−^	607.1219	2.4	7.2	545.1280 (8.0) (C_26_H_25_O_13_), 505.0990 (37.2) (C_23_H_21_O_13_), 463.0874 (47.5) (C_21_H_19_O_12_), 300.0268 (100) (C_15_H_8_O_7_^●^), 271.0235 (2.9) (C_14_H_7_O_6_), 255.0292 (1.5) (C_14_H_7_O_5_), 178.9978 (3.1) (C_8_H_3_O_5_), 151.0028 (2.6) (C_7_H_3_O_4_)	_	_	TA	[15]
23	7.00	Blumenin isomer (sesquiterpenoid cyclohexanone-hexuronosyl-hexoside)	C_25_H_40_O_13_	[M − H]^−^	547.2383	1.3	12.3	347.2062 (3.7) (C_19_H_31_O_7_), 209.1529 (1.7) (C_13_H_21_O_2_), 175.0239 (4.3) (C_6_H_7_O_6_), 161.0444 (1.5) (C_6_H_9_O_5_)	_	_	TA	[23]
24	7.17	Kaempferol-3-*O*-[HMG-(1→3/4)]-[dHex-(1→2)]-hexoside	C_33_H_38_O_19_	[M − H]^−^	737.1919	2.1	1.5	675.1922 (1.4) (C_32_H_35_O_16_), 635.1602 (5.7) (C_29_H_31_O_16_), 593.1528 (8.1) (C_27_H_29_O_15_), 429.0819 (0.7) (C_21_H_17_O_10_), 284.0317 (100) (C_15_H_8_O_6_^●^), 255.0293 (4.4) (C_14_H_7_O_5_), 227.0340 (2.0) (C_13_H_7_O_4_), 178.9974 (0.8) (C_8_H_3_O_5_), 151.0027 (0.9) (C_7_H_3_O_4_)	_	_	1.22 ^$^	[15]
25	7.19	Nicotiflorin (kaempferol-3-*O*-[Rha-(1→6)]-glucoside)	C_27_H_30_O_15_	[M − H]^−^	593.1501	1.8	4.4	327.0498 (2.4) (C_17_H_11_O_7_), 285.0392 (100) (C_15_H_9_O_6_), 255.0291 (1.9) (C_14_H_7_O_5_), 227.0344 (0.9) (C_13_H_7_O_4_), 151.0027 (1.2) (C_7_H_3_O_4_)	0.44	0.44		[14]
26	7.58	^#^ Narcissin (isorhamnetin-3-*O*-[α-Rha*p*-(1→6)]-β-glucoside)	C_28_H_32_O_16_	[M − H]^−^	623.1603	2.3	5.1	357.0609 (1.5) (C_18_H_13_O_8_), 315.0499 (100) (C_16_H_11_O_7_), 300.0266 (5.9) (C_15_H_8_O_7_^●^), 271.0244 (1.3) (C_14_H_7_O_6_), 151.0028 (0.7) (C_7_H_3_O_4_)	7.34	5.43	0.96	[14]
27	7.77	4,4′-Dimethoxy-2,2′-di-*O*-hexoside-truxinate (cyclodimer of GMCA)	C_32_H_40_O_18_	[M − H]^−^	711.2128	1.9	6. 9	549.1606 (12.9) (C_26_H_29_O_13_), 531.1497 (8.5) (C_26_H_27_O_12_), 369.0973 (51.4) (C_20_H_17_O_7_), 343.1181 (41.6) (C_19_H_19_O_6_), 299.1284 (23.7) (C_18_H_19_O_4_), 271.0970 (92.1), 256.0737 (2.7) (C_15_H_12_O_4_^●^), 193.0502 (100) (C_10_H_9_O_4_), 149.0599 (41.8) (C_9_H_9_O_2_), 134.0361 (8.2) (C_8_H_6_O_2_^●^)	_	TA	_	[15]
28	7.80	Kaempferol-3-*O*-[HMG-(1→3/4)]-hexoside	C_27_H_28_O_15_	[M − H]^−^	591.1344	1.9	5.6	529.1343 (13.8) (C_26_H_25_O_12_), 489.1031 (39.2) (C_23_H_21_O_12_), 447.0920 (31.8) (C_21_H_19_O_11_), 327.0499 (1.8) (C_17_H_11_O_7_), 284.0318 (100) (C_15_H_8_O_6_^●^), 255.0282 (1.4) (C_14_H_7_O_5_), 229.0503 (1.1) (C_13_H_9_O_4_), 151.0025 (0.9) (C_7_H_3_O_4_)	_	_	0.25	
29	7.81	Isorhamnetin-3-*O*-hexoside	C_22_H_22_O_12_	[M − H]^−^	477.1030	1.7	12.8	357.0604 (3.0) (C_18_H_13_O_8_), 314.0422 (100) (C_16_H_10_O_7_^●^), 299.0195 (1.46) (C_15_H_7_O_7_), 285.0397 (1.3) (C_15_H_9_O_6_), 271.0220 (1.6) (C_14_H_7_O_6_), 151.0034 (2.0) (C_7_H_3_O_4_)	TA	TA	TA	
30	9.10	^a^ Herniarin (7-methoxy- coumarin)	C_10_H_8_O_3_	[M + H]^+^	177.0557	−5.8	1.9	177.0537 (100), 133.0659 (4. 3), 121.0658 (2.9)	_	1.59	_	[15]
31	9.94	^#^ 7-*O*-Methylquercetin-3-*O*-[HMG-(1→6)]-[α-Rha-(1→2)]-β-galactoside	C_34_H_40_O_20_	[M − H]^−^	767.2035	0.7	17.0	705.2059 (0.4) (C_33_H_37_O_17_), 665.1726 (1.8) (C_30_H_33_O_17_), 623.1619 (4.8) (C_28_H_31_O_16_), 314.0429 (100) (C_16_H_10_O_7_^●^)_;_ 299.0196 (13.1) (C_15_H_7_O_7_), 271.0246 (2.4) (C_14_H_7_O_6_), 193.0145 (0.8) (C_9_H_5_O_5_), 165.0189 (1.5) (C_8_H_5_O_4_)	_	_	0.31	[15]
32	10.20	7-*O*-Methylkaempferol-3-*O*-[dHex-(1→2)]-hexoside	C_28_H_32_O_15_	[M − H]^−^	607.1672	−0.6	1.6	298.0484 (100) (C_16_H_10_O_6_^●^), 283.0248 (10.2) (C_15_H_7_O_6_), 271.0610 (0.9) (C_15_H_11_O_5_), 255.0298 (1.1) (C_14_H_7_O_5_), 165.0190 (0.7) (C_8_H_5_O_4_)	_	_	0.51	[15]
33	10.72	^#^ Oxytroflavoside A (7-*O*-methylkaempferol-3-*O*-[HMG-(1→6)]-[α-Rha-(1→2)]-β- galactoside)	C_34_H_40_O_19_	[M − H]^−^	751.2089	0.3	14.8	649.1772 (2.2) (C_30_H_33_O_16_), 607.1666 (4.4) (C_28_H_31_O_15_), 298.0481 (100) (C_16_H_10_O_6_^●^), 283.0247 (13.5) (C_15_H_7_O_6_); 271.0610 (0.9) (C_15_H_11_O_5_), 255.0298 (1.7) (C_14_H_7_O_5_), 165.0186 (0.7) (C_8_H_5_O_4_)	_	_	14.73	[15]
34	11.05	^#^ 7-*O*-Methylkaempferol-3-*O*-[HMG-(1→6)]-[α-Rha-(1→2)]-β-glucoside	C_34_H_40_O_19_	[M − H]^−^	751.2090	0.2	4.4	649.1788 (1.4) (C_30_H_33_O_16_), 607.1670 (5.5) (C_28_H_31_O_15_), 298.0483 (100) (C_16_H_10_O_6_^●^), 283.0247 (13.3) (C_15_H_7_O_6_), 271.0616 (0.8) (C1_5_H_11_O_5_), 255.0301 (1.5) (C_14_H_7_O_5_), 165.0188 (0.8) (C_8_H_5_O_4_)	_	_	0.91	[15]
35	11.26	^#^ Oxytroflavoside C (main) (7-*O*-methylkaempferol-3-*O*-[HMG-(1→4)]-[α-Rha-(1→2)]-β-galactoside]) Oxytroflavoside B (minor) (7-*O*-methylkaempferol-3-*O*-[HMG-(1→3)]-[α-Rha-(1→2)]-β-galactoside)	C_34_H_40_O_19_	[M − H]^−^	751.2086	0.6	8.1	649.1779 (1.6) (C_30_H_33_O_16_), 607.1676 (8.8) (C_28_H_31_O_15_), 298.0483 (100) (C_16_H_10_O_6_^●^), 283.0249 (11.2) (C_15_H_7_O_6_), 271.0614 (1.0) (C_15_H_11_O_5_), 255.0303 (1.2) (C_14_H_7_O_5_), 165.0189 (0.7) (C_8_H_5_O_4_)	_	_	1.38 ^$^	[15]
36												
37	11.54	7-*O*-Methyl-kaempferol-3-*O*-[HMG-(1→3/4)]-hexoside	C_28_H_30_O_15_	[M − H]^−^	605.1515	−0.5	11.4	543.1516 (8.0) (C_27_H_27_O_12_), 503.1198 (27.1) (C_24_H_23_O_12_), 461.1093 (22.9) (C_22_H_21_O_11_), 341.0663 (1.8) (C_18_H_13_O_7_), 298.0482 (100) (C_16_H_10_O_6_^●^), 283.0247 (6.2) (C_15_H_7_O_6_); 255.0302 (1.0) (C_14_H_7_O_5_), 165.0192 (1.3) (C_8_H_5_O_4_)	_	_	0.32	[15]
38	11.77	7-*O*-Methyl-kaempferol-3-*O*-[dHex-(1→2)]-pentoside	C_27_H_30_O_14_	[M − H]^−^	577.1565	−0.5	13.5	413.0888 (1.0) (C_21_H_17_O_9_), 298.0490 (100) (C_16_H_10_O_6_^●^), 283.0250 (7.3) (C_15_H_7_O_6_), 255.0302 (1.0) (C_14_H_7_O_5_), 165.0188 (0.9) (C_8_H_5_O_4_)	_	_	0.12	[15]
		Sum							12.95	24.03	29.56	

Notes: ^a^ Identification with an analytical standard. ^#^ Isolated and NMR confirmed. * Accuracy of mass measurements expressed in parts per million (ppm). ** Isotopic pattern fit factor (mσ). %—Percentage of a compound in the purified extracts. ^●^ Anionradical. ^$^ Co-eluting peaks. CQA—caffeoylquinic acid; *p*CoQA—*p*-coumaroylquinic acid; FerQA—feruloylquinic acid; *p*CoA—*p*-coumaric acid; dHex—deoxyhexosyl; Rha—rhamnopyranosyl; Api—apifuranosyl; HMG—3-hydroxy-3-methylglutaryl; TA—trace amount; NC—not calculated; Ref.—references.

**Table 2:** Determination of antioxidant properties of the examined *Herniaria glabra* (HG-PE), *H. polygama* (HP-PE), and *H. incana* herb (HIh-PE) purified extracts, based on measurements of thiobarbituric acid-reactive substances (TBARS) and protein thiol groups levels as well as on the ferric reducing ability of blood plasma (FRAP), under the ONOO^−^-induced oxidative stress In Vitro. Statistical significance: ^#^ *p* < 0.05, ^##^ *p* < 0.001 plasma treated with ONOO^−^ in the absence of examined purified extracts vs. control plasma; * *p* < 0.05, ** *p* < 0.01 plasma treated with ONOO^−^ in the absence of examined purified extracts vs. plasma treated with ONOO^−^ in the presence of examined substances.

	(µg/ mL)	TBARS (nmol/mL of Plasma) (*n* = 9)	Protein-SH Groups (µmol/mL of Plasma) (*n* = 8)	FRAP (mM Fe^2+^) (*n* = 9)
Control (untreated) plasma	0	0.078 ± 0.011	0.335 ± 0.019	0.460 ± 0.035
Plasma treated with ONOO^−^	0	0.122 ± 0.015 ^#^	0.229 ± 0.029 ^##^	0.380 ± 0.027 ^##^
Trolox	1	0.104 ± 0.027	0.267 ± 0.025 *	0.422 ± 0.031
	5	0.108 ± 0.027	0.274 ± 0.029 **	0.462 ± 0.075 **
	50	0.106 ± 0.025	0.288 ± 0.038 **	0.603 ± 0.060 **
HG-PE	1	0.100 ± 0.022 *	0.260 ± 0.026 *	0.418 ± 0.033
	5	0.099 ± 0.023 **	0.268 ± 0.022 **	0.429 ± 0.028 *
	50	0.102 ± 0.022 **	0.262 ± 0.024 **	0.447 ± 0.040 *
HP-PE	1	0.101 ± 0.022 *	0.261 ± 0.023 *	0.428 ± 0.029
	5	0.093 ± 0.019 **	0.264 ± 0.029 *	0.442 ± 0.033 **
	50	0.100 ± 0.026 *	0.258 ± 0.027 *	0.458 ± 0.032 **
HIh-PE	1	0.096 ± 0.026 *	0.258 ± 0.031 *	0.425 ± 0.033
	5	0.101 ± 0.024	0.262 ± 0.024 **	0.440 ± 0.028 *
	50	0.099 ± 0.022 *	0.264 ± 0.025 **	0.485 ± 0.091 **

**Table 3:** The influence of the examined *Herniaria glabra* (HG-PE), *H. polygama* (HP-PE), and *H. incana* herb (HIh-PE) purified extracts on the viability of peripheral blood mononuclear cells (PBMCs). The viability of control (untreated) PBMCs was assumed as 100%; ** *p* < 0.01.

The Examined Extracts	(μg/mL)	PBMCs Viability (%)	
		the Resazurin-Based Assay (*n* = 7)	the Trypan Blue Excluding Test (*n* = 11)
HG-PE	5	93.47 ± 6.83	102.73 ± 11.15
	25	95.42 ± 6.82	97.49 ± 8.25
	50	93.82 ± 12.51	89.22 ± 14.37
HP-PE	5	93.11 ± 10.27	99.06 ± 11.36
	25	95.17 ± 8.46	97.83 ± 10.97
	50	68.49 ± 17.29 **	87.69 ± 12.08
HIh-PE	5	93.52 ± 10.47	101.18 ± 13.86
	25	96.85 ± 9.89	101.76 ± 13.29
	50	99.98 ± 13.60	92.84 ± 14.54

## Data Availability

The data supporting the results are present in the manuscript.

## Sample Availability

Samples of the plant materials and the isolated compounds **9**–**11**, **13**, **15**, **16**, **18**, **26**, **31**, **33**–**36** and kaempferol, as well as commercial standards **4**, **6**, **30,** are available from the authors.
